# Long noncoding RNA B3GALT5-AS1 suppresses colon cancer liver metastasis via repressing microRNA-203

**DOI:** 10.18632/aging.101628

**Published:** 2018-12-10

**Authors:** Liang Wang, Zhewei Wei, Kaiming Wu, Weigang Dai, Changhua Zhang, Jianjun Peng, Yulong He

**Affiliations:** 1 Department of Gastrointestinal Surgery, The First Affiliated Hospital, Sun Yat-sen University, Guangzhou 510080, China; * Equal contribution

**Keywords:** long noncoding RNA, colon cancer, liver metastasis, microRNA, epithelial-to-mesenchymal transition

## Abstract

Long noncoding RNAs (lncRNAs) are implicated in various cancers, including colon cancer. Liver metastasis is the main cause of colon cancer-related death. However, the roles of lncRNAs in colon cancer liver metastasis are still largely unclear. In this study, we identified a novel lncRNA B3GALT5-AS1, which is reduced in colon cancer tissues and further reduced in colon cancer liver metastasis tissues. Reduced expression of B3GALT5-AS1 is associated with liver metastasis and poor outcome of colon cancer patients. Gain-of-function and loss-of-function assays revealed that B3GALT5-AS1 inhibited proliferation but promoted migration and invasion of colon cancer cells. Further investigation revealed that B3GALT5-AS1 directly bound to the promoter of *miRNA-203*, repressed *miR-203* expression, upregulated miR-203 targets ZEB2 and SNAI2, and induced epithelial-to-mesenchymal transition (EMT). *In vivo* study revealed that B3GALT5-AS1 suppressed colon cancer liver metastasis via its binding on *miR-203* promoter and the repression of miR-203. miR-203 is increased and epithelial phenotype is preferred in colon cancer liver metastasis tissues. Collectively, our data revealed the suppressive roles of B3GALT5-AS1/miR-203/EMT regulation axis in colon cancer liver metastasis. Our data suggested that the activating B3GALT5-AS1/miR-203/EMT axis may be potential therapeutic strategy for colon cancer liver metastasis.

## Introduction

Colon cancer is one of the most prevalent malignancies and causes of cancer-related deaths worldwide [1]. Distant metastasis, especially liver metastasis accounts for the major cause of deaths of colon cancer patients [2]. Most colon cancer patients with liver metastasis are not suitable for surgery [3]. In addition, there is a lack of effective treatments for colon cancer patients with liver metastasis [4]. Thus, the prognoses of colon cancers with liver metastases are very poor with a 5-year survival rate of 10-15% [5]. Therefore, further revealing critical molecular mechanisms driving colon cancer liver metastasis and developing more effective therapies for colon cancers with liver metastasis are urgently needed.

Colon cancer liver metastasis is a complex and multistep process [6]. Many molecules are contradictorily involved in this process [7,8]. The detailed molecular mechanisms mediating the process are largely unclear [9]. Epithelial-to-mesenchymal transition (EMT) plays critical roles during the process of colon liver metastasis [10,11]. EMT permits the migration and invasion of various tumor cells, which is beneficial for the early invasion of primary cancers [12]. EMT may also discount proliferative capacity of cancer cells [13]. In the distant metastatic locations, disseminated cancer cells require mesenchymal-to-epithelial transition (MET), a reverse process of EMT, to settle and growth [7]. MET enables metastatic cancer cells to acquire epithelial phenotype and colonize in distant organs [14–16]. Epithelial marker E-cadherin is reported to be elevated in lymph node metastases and distant metastases relative to primary tumors [17]. Therefore, identifying critical EMT-MET regulators during colon cancer liver metastasis cascade are beneficial for appropriate therapy of colon cancer liver metastasis.

Genomic and transcriptomic sequencings have demonstrated that although 70% of human genome transcribe RNA molecules, only about 2% of human genome encode proteins [18]. Therefore, most of human transcriptome are non-coding RNAs [19]. Many of these non-coding RNAs have critical regulatory roles in cancers [20]. Long noncoding RNA (lncRNA) is a class of RNA transcript with limited protein coding ability and greater than 200 nucleotides in length [21]. Accumulating evidences displayed that lncRNAs are commonly deregulated in many pathological states and have important roles during various pathophysiological processes [22–27]. As to colon cancer, several lncRNAs are revealed to regulate colon cancer cells proliferation, apoptosis, migration, invasion, chemoresistance, and so on, such as lncRNA N-BLR, GAS5, HNF1A-AS1, CRNDE, LINC01133 [28–32]. However, the roles of lncRNAs in EMT and liver metastasis of colon cancer are largely unclear.

microRNA (miRNA) is another class of non-coding RNA transcript with 20-25 nucleotides in length [33]. Similarly, miRNAs are reported to have important regulatory roles during various pathophysiological processes [34–38]. Several miRNAs are well-known EMT regulators through repressing EMT-inducing transcription factors [39,40]. miR-200 family have been reported to inhibit EMT by directly repressing ZEB1 and ZEB2 [39,41–43]. miR-203 has been reported to inhibit EMT via repressing ZEB2 and SNAI2 [44,45]. However, miR-203 has different roles in different cancers [40,46–49]. miR-203 is revealed to exert tumor suppressive roles in prostate, lung, nasopharyngeal, and colorectal cancer [50–52]. However, miR-203 is also revealed to be increased in colorectal cancer tissues compared with adjacent normal mucosa [46]. Furthermore, miR-203 is also revealed to be upregulated in colorectal cancer liver metastasis tissues compared with primary colorectal cancer tissues [46]. Meta-analysis indicated that the upregulation of miR-203 indicted worse prognosis in colorectal cancer [49]. These controversial results suggested that more investigations of the expression and roles of miR-203 in EMT and liver metastasis of colon cancer are needed.

In this study, using public available RNA-seq dataset of colon cancer [53], we identified that lncRNA B3GALT5-AS1 is reduced in colon cancer tissues, and further reduced in colon cancer liver metastasis tissues. In clinical specimens, we further confirmed the expression pattern of B3GALT5-AS1 in colon cancer and liver metastasis. Furthermore, we confirmed the negative correlation between miR-203 and B3GALT5-AS1 expression pattern in colon cancer liver metastasis. In addition, biological roles of B3GALT5-AS1 and miR-203 in EMT and liver metastasis of colon cancer were explored using gain-of-function and loss-of-function experiments.

## RESULTS

### B3GALT5-AS1 is reduced in colon cancer and further reduced in liver metastasis tissues

**Figure 1:**
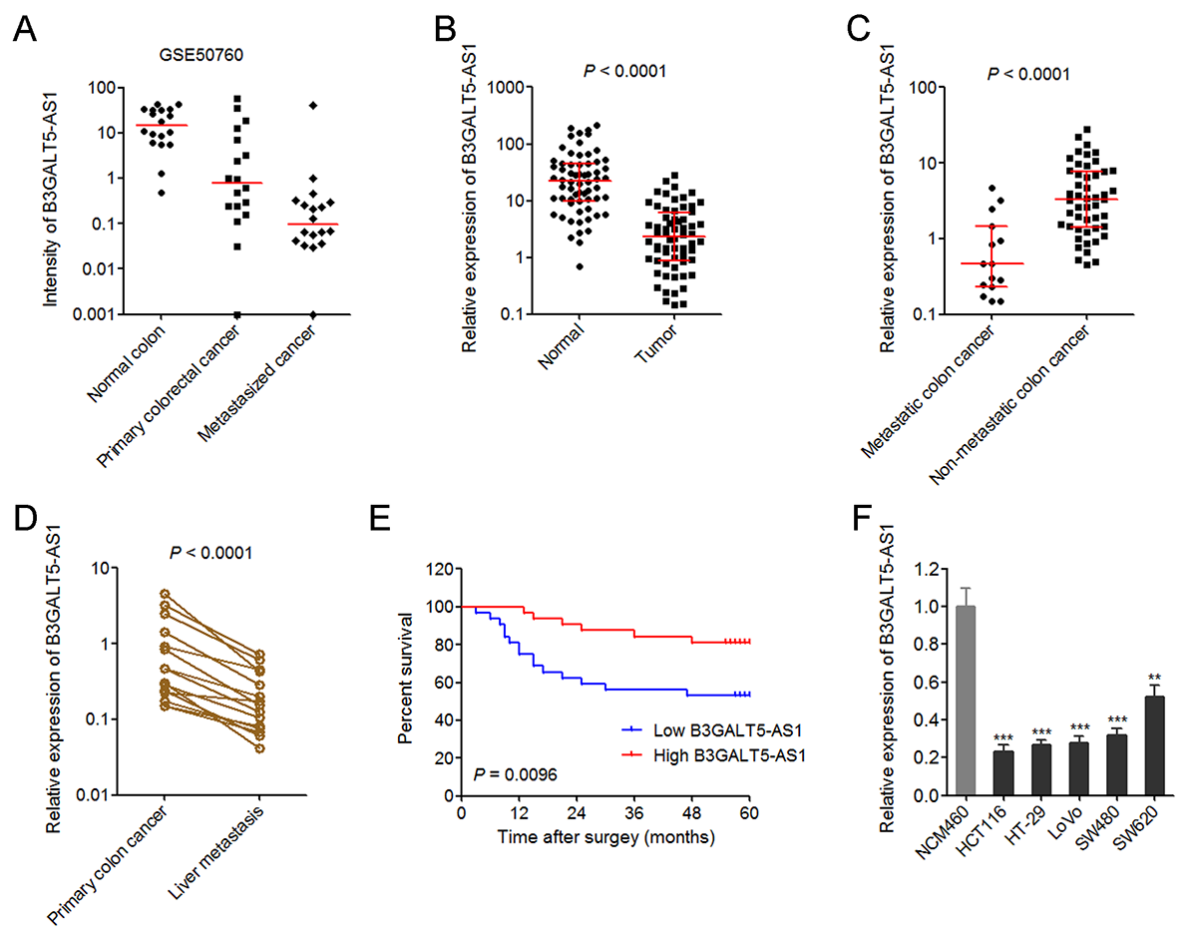
**The expression pattern of B3GALT5-AS1 in colon cancer and its association with prognosis.** (**A**) The expression intensity of B3GALT5-AS1 in 18 pairs of normal colonic epithelium, primary colorectal cancers, and metastasized cancers in liver from GSE50760. (**B**) The expression of B3GALT5-AS1 in 64 pairs of primary colon cancer tissues and adjacent colonic epithelium tissues was detected using qRT-PCR. *P* < 0.0001, Wilcoxon signed-rank test. (**C**) The expression of B3GALT5-AS1 in 15 colon cancer tissues with metastasis and 49 colon cancer tissues without metastasis. *P* < 0.0001, Mann-Whitney test. (**D**) The expression of B3GALT5-AS1 in 15 pairs of primary colon cancer tissues and corresponding liver metastasis tissues was measured using qRT-PCR. *P* < 0.0001, Wilcoxon signed-rank test. (**E**) Kaplan-Meier survival analysis of the correlation between B3GALT5-AS1 expression level and overall survival of 64 colon cancer patients. The median expression level of B3GALT5-AS1 was used as cut-off. *P* = 0.0096, Log-rank test. (**F**) The expression of B3GALT5-AS1 in normal colonic epithelial cell line NCM460 and colon cancer cell lines HCT116, HT-29, LoVo, SW480 and SW620 was measured using qRT-PCR. Results are displayed as mean ± s.d. of three independent experiments. **P < 0.01, ***P < 0.001, Student’s *t*-test.

Analyzing the RNA-seq dataset from GSE50760 which containing 18 normal colonic epithelium, 18 primary colorectal cancers, and 18 metastasized cancers in liver, we noted that lncRNA B3GALT5-AS1 (C21orf88) is reduced in primary colorectal cancers compared with normal colonic epithelium and is further reduced in metastasized cancers in liver (Fig. 1A). To further explore B3GALT5-AS1 expression pattern in human colon cancer, we collected 64 pairs of primary colon cancer tissues and corresponding adjacent colonic epithelium tissues. Through searching the National Center for Biotechnology Information (NCBI), we found two transcript variants of B3GALT5-AS1. qRT-PCR results displayed that transcript variant 2 (NCBI Reference Sequence: NR_026543.1) is the main transcript of B3GALT5-AS1 in both normal colon tissues and colon cancer tissues (Figure S1). Furthermore, transcript variant 2 is reduced in colon cancer, and while transcript variant 1 doesn’t have significant difference between colon cancer and normal colonic epithelium tissues (Figure S1). Therefore, we focused our attention on transcript variant 2 of B3GALT5-AS1.

The expression of B3GALT5-AS1 in 64 pairs of primary colon cancer tissues and corresponding normal colonic epithelium tissues was measured via qRT-PCR. As displayed in Fig. 1B, B3GALT5-AS1 is markedly reduced in primary colon cancer tissues compared with colonic epithelium tissues. Analyses of the association between the expression of B3GALT5-AS1 and clinicopathologic features of colon cancers displayed that lower B3GALT5-AS1 expression is correlated with larger tumor size, distant metastasis, and advanced AJCC stages (Table 1). To confirm the association between B3GALT5-AS1 expression and distant metastasis, we re-analyzed the expression of B3GALT5-AS1 in colon cancer tissues with (n = 15) or without (n = 49) metastasis. The results displayed that B3GALT5-AS1 is significantly reduced in primary colon cancer tissues with metastasis compared with that without metastasis (Fig. 1C). For these 15 colon cancers with metastasis, we collected their corresponding liver metastasis tissues. The expression of B3GALT5-AS1 in these 15 pairs of primary colon cancer tissues and corresponding liver metastasis tissues was measured via qRT-PCR. As displayed in Fig. 1D, B3GALT5-AS1 is markedly reduced in liver metastasis tissues compared with primary colon cancer tissues. Kaplan-Meier survival analysis of these 64 colon cancer patients displayed that colon cancer patients with lower B3GALT5-AS1 expression had worse survival than those with higher B3GALT5-AS1 expression (Fig. 1E). Moreover, the expression of B3GALT5-AS1 in normal colonic epithelial cell line NCM460 and colon cancer cell lines HCT116, HT-29, LoVo, SW480 and SW620 was measured by qRT-PCR. As displayed in Fig. 1F, B3GALT5-AS1 was also significantly reduced in colon cancer cell lines compared with normal colonic epithelial cell line. These data suggested that B3GALT5-AS1 is reduced in colon cancer and further reduced in liver metastasis tissues. Low expression of B3GALT5-AS1 indicts poor outcome of colon cancers.

**Table 1:** Correlation between B3GALT5-AS1 expression and clinicopathologic features of colon cancers.

Features	N of cases	B3GALT5-AS1		*P*-value
		Low	High	
Total	64	32	32	
Age (years)				0.316
>65	34	19	15	
≤65	30	13	17	
Gender				0.802
Male	31	15	16	
Female	33	17	16	
Location				0.802
Right	35	17	18	
Left	29	15	14	
Tumor size (cm)				**0.002**
>3	36	24	12	
≤3	28	8	20	
Depth of invasion				0.757
T1	2	1	1	
T2	9	6	3	
T3	25	12	13	
T4	28	13	15	
Lymph node metastasis				0.523
N0	25	12	13	
N1	22	13	9	
N2	17	7	10	
Distant metastasis				**0.008**
M0	49	20	29	
M1	15	12	3	
AJCC stage				**0.043**
I	8	4	4	
II	17	8	9	
III	24	8	16	
IV	15	12	3	

The median expression level of B3GALT5-AS1 was used as cut-off.

*P*-value was acquired by Pearson chi-square tests.

### B3GALT5-AS1 suppresses colon cancer cell proliferation

**Figure 2:**
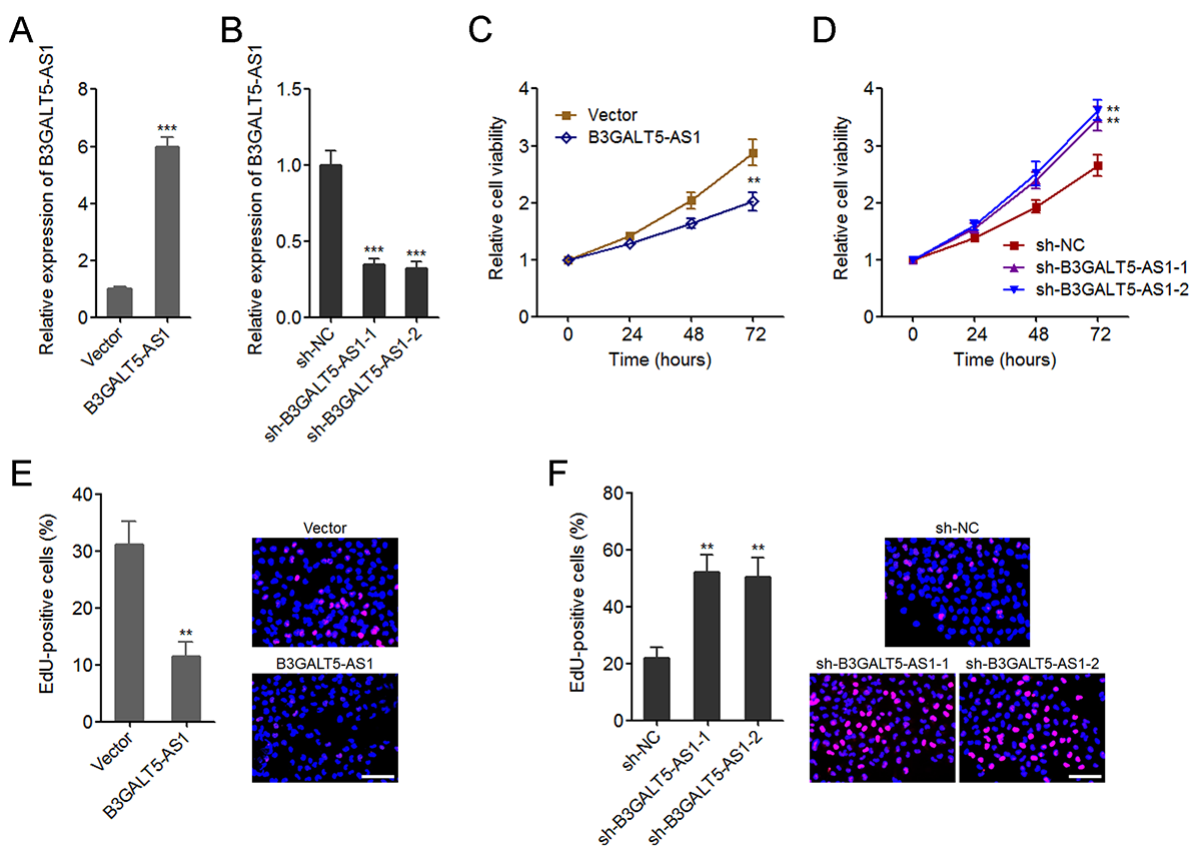
**B3GALT5-AS1 suppressed colon cancer cell proliferation.** (**A**) The expression of B3GALT5-AS1 in B3GALT5-AS1 stably overexpressed and control HCT116 cells was detected using qRT-PCR. (**B**) The expression of B3GALT5-AS1 in B3GALT5-AS1 stably depleted and control SW620 cells was detected using qRT-PCR. (**C**) Cell viability of B3GALT5-AS1 stably overexpressed and control HCT116 cells was detected using Glo cell viability assay. (**D**) Cell viability of B3GALT5-AS1 stably depleted and control SW620 cells was detected using Glo cell viability assay. (**E**) Cell proliferation of B3GALT5-AS1 stably overexpressed and control HCT116 cells was detected using EdU incorporation assay. The red color indicts EdU-positive cells. Scale bars = 100 μm. (**F**) Cell proliferation of B3GALT5-AS1 stably depleted and control SW620 cells was detected using EdU incorporation assay. The red color indicts EdU-positive cells. Scale bars = 100 μm. Results are displayed as mean ± s.d. of three independent experiments. **P < 0.01, ***P < 0.001, Student’s *t*-test.

To explore the effects of B3GALT5-AS1, we stably overexpressed B3GALT5-AS1 in HCT116 cells by transfecting B3GALT5-AS1 overexpression plasmid (Fig. 2A), and stably knocked-down B3GALT5-AS1 in SW620 cells by transfecting two independent shRNAs against B3GALT5-AS1 (Fig. 2B). Glo cell viability assays displayed that B3GALT5-AS1 overexpression markedly reduced cell viability, and while B3GALT5-

AS1 knockdown significantly upregulated cell viability of colon cancer cells (Fig. 2C, D). EdU incorporation assays further displayed that enhanced expression of B3GALT5-AS1 markedly suppressed cell proliferation, and while B3GALT5-AS1 knockdown markedly promoted proliferation of colon cancer cells (Fig. 2E, F). Due to B3GALT5-AS1 is high expressed in normal colonic epithelial cell line NCM460, we further determined the effects of B3GALT5-AS1 knockdown on NCM460 cell viability and cell proliferation using Glo cell viability assay and EdU incorporation assay. As displayed in Figure S2A-C, transient knockdown of B3GALT5-AS1 also promoted NCM460 cell proliferation. Collectively, these data suggested that B3GALT5-AS1 suppresses cell viability and cell proliferation of colon cancer and colonic epithelial cells.

### B3GALT5-AS1 promotes colon cancer cell migration, invasion and EMT

**Figure 3:**
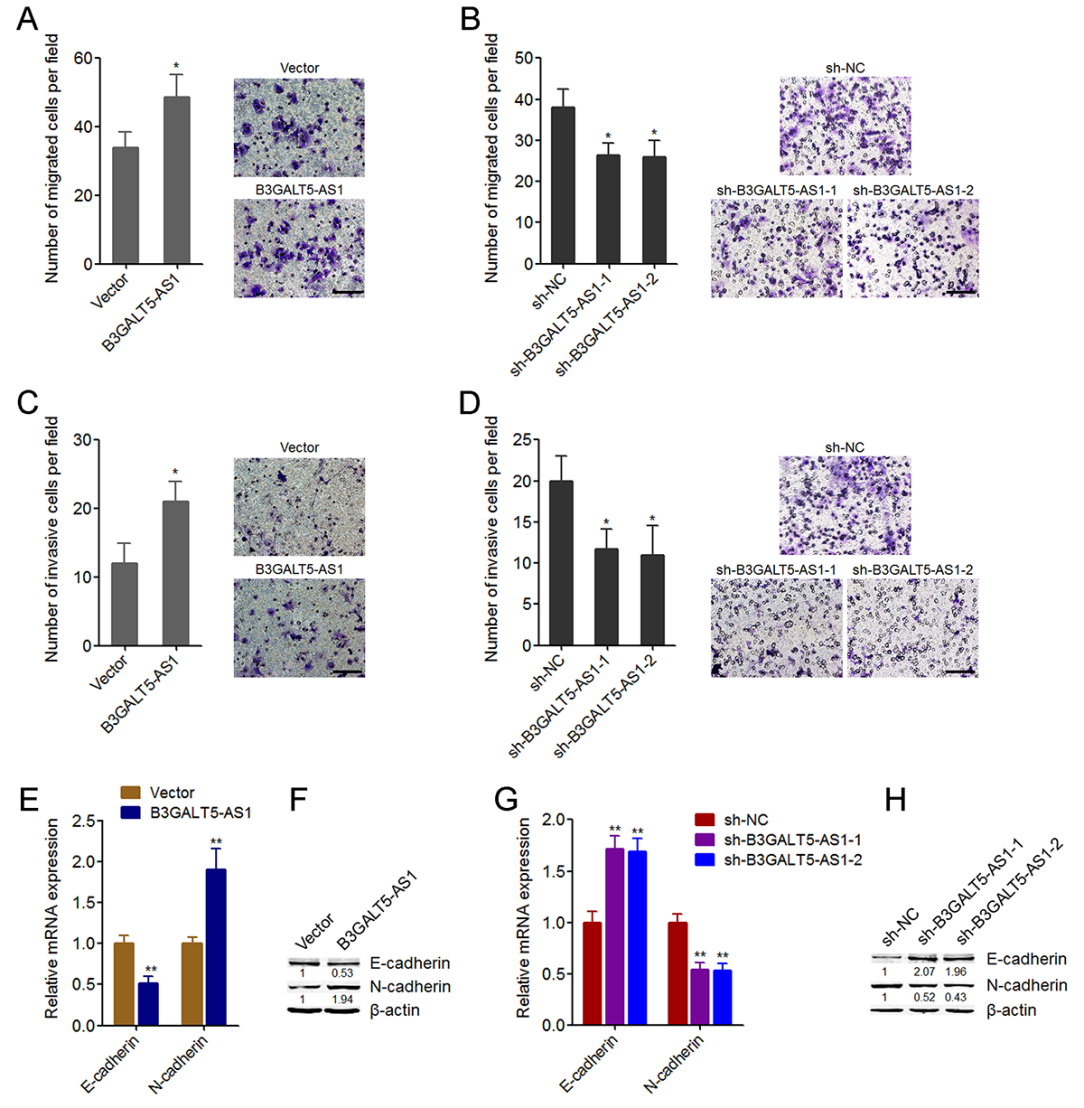
**B3GALT5-AS1 promoted migration, invasion, and EMT of colon cancer cells.** (**A**) Cell migration of B3GALT5-AS1 stably overexpressed and control HCT116 cells was detected using transwell migration assay. Scale bars = 100 μm. (**B**) Cell migration of B3GALT5-AS1 stably depleted and control SW620 cells was detected using transwell migration assay. Scale bars = 100 μm. (**C**) Cell invasion of B3GALT5-AS1 stably overexpressed and control HCT116 cells was detected using transwell invasion assay. Scale bars = 100 μm. (**D**) Cell invasion of B3GALT5-AS1 stably depleted and control SW620 cells was detected using transwell invasion assay. Scale bars = 100 μm. (**E**) E-cadherin and N-cadherin mRNA levels in B3GALT5-AS1 stably overexpressed and control HCT116 cells were detected using qRT-PCR. (**F**) E-cadherin and N-cadherin protein levels in B3GALT5-AS1 stably overexpressed and control HCT116 cells were detected using western blot. (**G**) E-cadherin and N-cadherin mRNA levels in B3GALT5-AS1 stably depleted and control SW620 cells were detected using qRT-PCR. (**H**) E-cadherin and N-cadherin protein levels in B3GALT5-AS1 stably depleted and control SW620 cells were detected using western blot. Results are displayed as mean ± s.d. of three independent experiments. *P < 0.05, **P < 0.01, Student’s *t*-test.

We then further explored the roles of B3GALT5-AS1 in migration and invasion of colon cancer cells. Transwell migration assays displayed that B3GALT5-AS1 overexpression promoted cell migration, and while B3GALT5-AS1 knockdown inhibited cell migration of colon cancer cells (Fig. 3A, B). Transwell invasion assays displayed that enhanced expression of B3GALT5-AS1 promoted invasion, and while B3GALT5-AS1 knockdown repressed invasion of colon cancer cells (Fig. 3C, D). Similarly, B3GALT5-AS1 knockdown also repressed migration and invasion of NCM460 cells (Figure S2D, E). The opposing effects of B3GALT5-AS1 on cell proliferation and migration, invasion implied that EMT may mediate the roles of B3GALT5-AS1 in colon cancer. Thus, we further explored the roles of B3GALT5-AS1 in EMT of colon cancer cells. The results displayed that B3GALT5-AS1 overexpression reduced the expression of epithelial marker E-cadherin and increased mesenchymal marker N-cadherin (Fig. 3E, F), suggesting that B3GALT5-AS1 overexpression induced EMT of colon cancer cells. B3GALT5-AS1 knockdown upregulated the expression of E-cadherin and downregulated N-cadherin (Fig. 3G, H), suggesting that B3GALT5-AS1 knockdown repressed EMT of colon cancer cells. Similarly, B3GALT5-AS1 knockdown also repressed EMT of NCM460 cells (Figure S2F). These data demonstrated that B3GALT5-AS1 promotes migration, invasion and EMT of colon cancer and colonic epithelial cells.

### B3GALT5-AS1 directly binds the promoter of *miR-203* and represses the expression of *miR-203*

**Figure 4:**
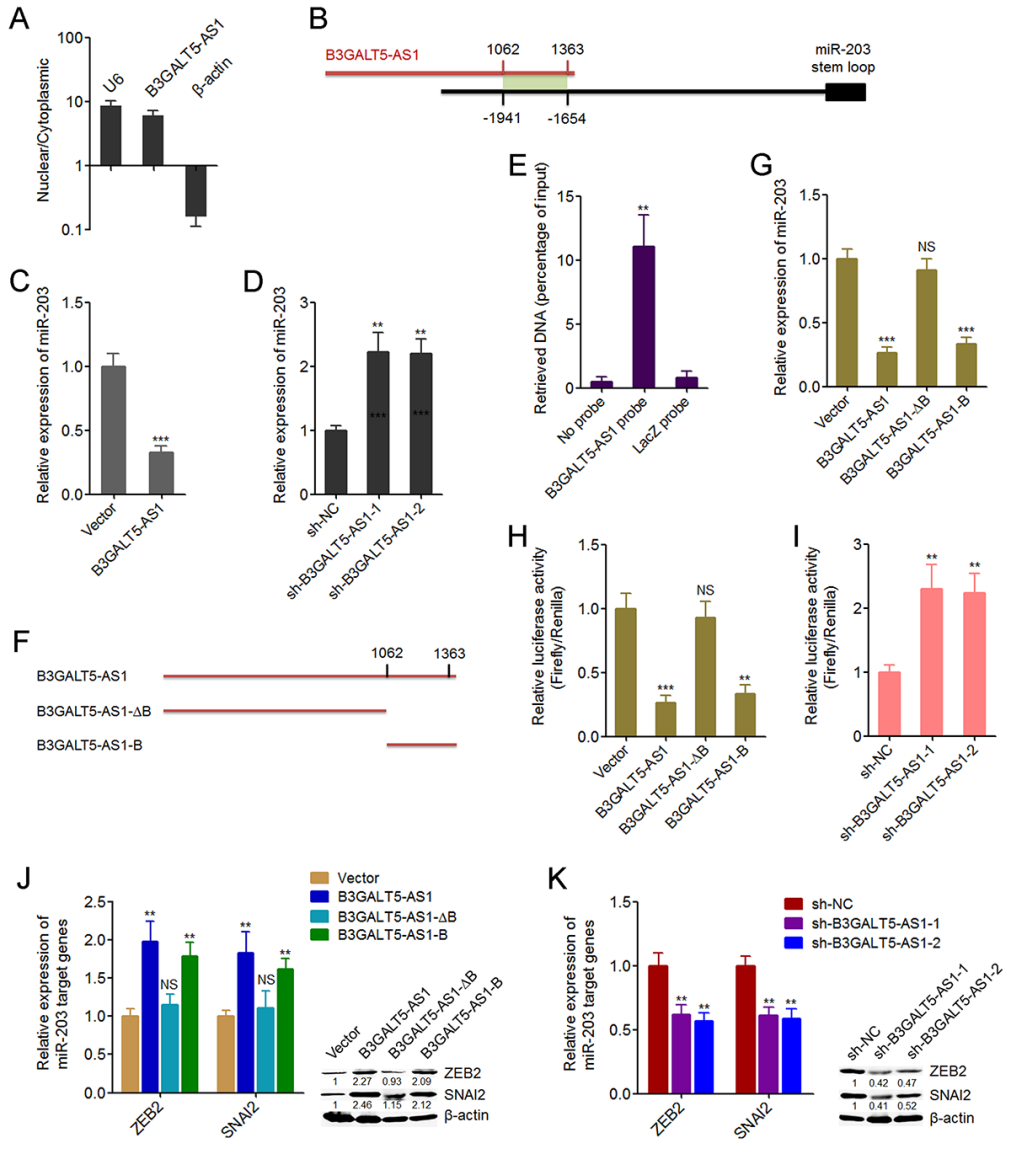
**B3GALT5-AS1 bound to the promoter of *miR-203* and repressed the expression of miR-203.** (**A**) The subcellular distribution of B3GALT5-AS1 in the cytoplasmic and nuclear fractions of HCT116 cells was evaluated using cytoplasmic and nuclear RNA isolation followed by qRT-PCR.β-actin and U6 were used as cytoplasmic and nuclear controls, respectively. (**B**) Schematic outline of the predicted interaction sites between B3GALT5-AS1 and the promoter of *miR-203*. (**C**) The expression of miR-203 in B3GALT5-AS1 stably overexpressed and control HCT116 cells was detected using qRT-PCR. (**D**) The expression of miR-203 in B3GALT5-AS1 stably depleted and control SW620 cells was detected using qRT-PCR. (**E**) ChIRP assays in HCT116 cells were carried out with anti-sense probe sets specific for B3GALT5-AS1 or LacZ (negative control). The enriched DNA was measured using qRT-PCR with specific primers against *miR-203* promoter. (**F**) Schematic outline of the constructed different depletion transcripts of B3GALT5-AS1. (**G**) After transient transfections of the different B3GALT5-AS1 expressing plasmids into HCT116 cells, miR-203 expression was measured using qRT-PCR. (**H**) After transient co-transfection of the firefly luciferase reporter containing the promoter of *miR-203*, renilla luciferase expression plasmid pRL-TK, and the different B3GALT5-AS1 expression plasmids into HCT116 cells, luciferase activities were detected using dual luciferase reporter assays. Results are displayed as the relative ratio of firefly luciferase activity to renilla luciferase activity. (**I**) After transient co-transfection of the firefly luciferase reporter containing the promoter of *miR-203* and pRL-TK into B3GALT5-AS1 stably depleted and control SW620 cells, luciferase activities were measured by dual luciferase reporter assays. Results are shown as the relative ratio of firefly luciferase activity to renilla luciferase activity. (**J**) After transient transfections of the different B3GALT5-AS1 expressing plasmids into HCT116 cells, the expression of ZEB2 and SNAI2 was detected using qRT-PCR and western blot. (**K**) The expression of ZEB2 and SNAI2 in B3GALT5-AS1 stably depleted and control SW620 cells was detected using qRT-PCR and western blot. Data are displayed as mean ± s.d. of three independent experiments. **P < 0.01, ***P < 0.001, NS, not significant, Student’s *t*-test.

To investigate the underpinning mechanism mediating the roles of B3GALT5-AS1 in colon cancer, we first confirmed the subcellular distribution of B3GALT5-AS1 in colon cancer cells using cytoplasmic and nuclear RNA purification. As displayed in Fig. 4A, B3GALT5-AS1 was dominantly localized in the nucleus. Several miRNAs are reported to be involved in EMT [11]. miR-203, miR-200 family (including miR-200a, miR-200b, miR-200c, miR-141, miR-429), miR-34a, miR-9 are reported to inhibit EMT [11,44]. miR-29a is reported to promote EMT. Therefore, we predicted the potential roles of B3GALT5-AS1 on these miRNAs via searching the potential binding sites of B3GALT5-AS1 on the promoters of these miRNAs using Basic Local Alignment Search Tool (BLAST) (https://blast.ncbi.nlm.nih.gov/Blast.cgi). As displayed in Figure S3, the promoter of *miR-203* has the strongest binding potential with B3GALT5-AS1. The predicted interaction region covers 1062-1363 nucleotides of B3GALT5-AS1 (Fig. 4B). Then, we investigated whether B3GALT5-AS1 regulates miR-203 expression in colon cancer cells. qRT-PCR results displayed that B3GALT5-AS1 overexpression significantly suppressed miR-203 expression, and while depletion of B3GALT5-AS1 markedly upregulated miR-203 expression (Fig. 4C, D). ChIRP assays displayed that *miR-203* promoter was specifically enriched by B3GALT5-AS1 antisense probe sets (Fig. 4E). Next, we expressed the truncated B3GALT5-AS1 fragments with or without the binding sites, which encode 1062-1363 nucleotides or 1-1061 nucleotides of B3GALT5-AS1, respectively (Fig. 4F). Transient transfections of the full-length or truncated B3GALT5-AS1 expression plasmids into HCT116 cells revealed that the depletion of the binding sites abolished the repressive roles of B3GALT5-AS1 on miR-203 expression, and while only the binding sites of B3GALT5-AS1 could sufficiently repress miR-203 expression (Fig. 4G). These results suggested that the binding region is responsible for the effects of B3GALT5-AS1 on miR-203. To further investigate whether B3GALT5-AS1 regulates the promoter activity of *miR-203*, we cloned *miR-203* promoter containing the binding region into luciferase reporter. Dual luciferase reporter assays displayed that B3GALT5-AS1 overexpression significantly downregulated the promoter activity of *miR-203*, which was abolished by the depletion of binding sites of B3GALT5-AS1, and while only the binding sites of B3GALT5-AS1 could sufficiently downregulated *miR-203* promoter activity (Fig. 4H). Conversely, B3GALT5-AS1 knockdown significantly upregulated *miR-203* promoter activity (Fig. 4I). miR-203 is reported to inhibit EMT via repressing the expression of EMT-inducing transcription factor ZEB2 and SNAI2 [44,45]. Therefore, we further investigate the roles of B3GALT5-AS1 on miR-203 targets ZEB2 and SNAI2. Transient transfections of the different B3GALT5-AS1 expression plasmids into HCT116 cells demonstrated that B3GALT5-AS1 overexpression upregulated ZEB2 and SNAI2, which were abolished by the depletion of binding sites of B3GALT5-AS1, and while only the binding sites of B3GALT5-AS1 could sufficiently upregulated ZEB2 and SNAI2 (Fig. 4J). Conversely, B3GALT5-AS1 knockdown downregulated ZEB2 and SNAI2 (Fig. 4K). All these results suggested that B3GALT5-AS1 inhibited miR-203 and upregulated miR-203 targets ZEB2 and SNAI2 via interacting with *miR-203* promoter.

### miR-203 is increased in colon cancer and further increased in liver metastasis

**Figure 5:**
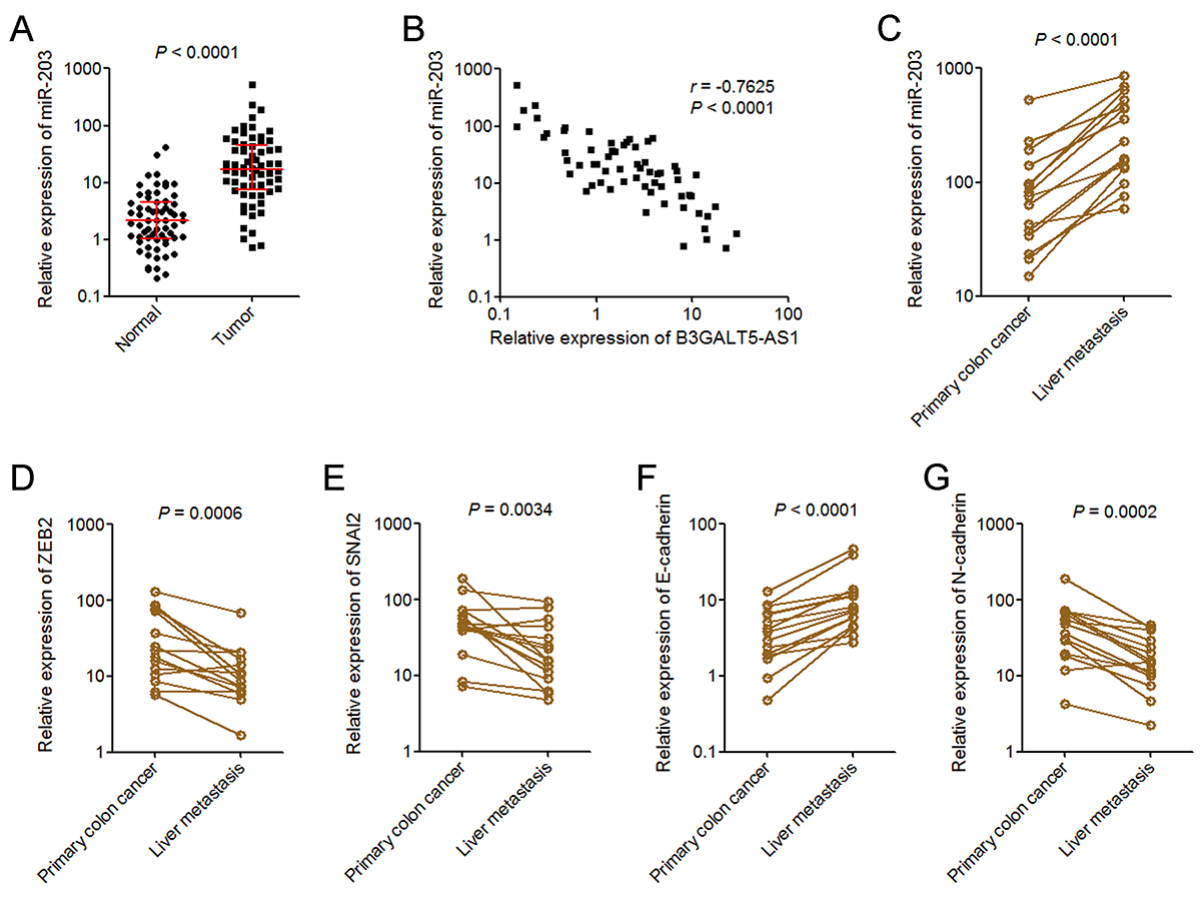
**miR-203 expression pattern in colon cancer.** (**A**) miR-203expression in 64 pairs of primary colon cancer tissues and adjacent colonic epithelium tissues was measured by qRT-PCR. *P* < 0.0001, Wilcoxon signed-rank test. (**B**) The correlation between B3GALT5-AS1 and miR-203 expression level in colon cancer tissues. *n* = 64, *r* = -0.7625, *P* < 0.0001, Pearson’s correlation analysis. (**C**) The expression of miR-203 in 15 pairs of primary colon cancer tissues and corresponding liver metastasis tissues was measured using qRT-PCR. *P* < 0.0001, Wilcoxon signed-rank test. (**D**) The expression of ZEB2 in 15 pairs of primary colon cancer tissues and corresponding liver metastasis tissues was measured using qRT-PCR. *P* = 0.0006, Wilcoxon signed-rank test. (**E**) The expression of SNAI2 in 15 pairs of primary colon cancer tissues and corresponding liver metastasis tissues was measured using qRT-PCR. *P* = 0.0034, Wilcoxon signed-rank test. (**F**) The expression of E-cadherin in 15 pairs of primary colon cancer tissues and corresponding liver metastasis tissues was measured using qRT-PCR. *P* < 0.0001, Wilcoxon signed-rank test. (**G**) The expression of N-cadherin in 15 pairs of primary colon cancer tissues and corresponding liver metastasis tissues was measured using qRT-PCR. *P* = 0.0002, Wilcoxon signed-rank test.

To explore whether the regulation of miR-203 and EMT by B3GALT5-AS1 exists *in vivo*, we measured miR-203 expression in the same 64 pairs of primary colon cancer tissues and corresponding adjacent colonic epithelium tissues used in Fig. 1B. As displayed in Fig. 5A, miR-203 was markedly upregulated in colon cancer tissues compared with colonic epithelium tissues. Analyses of the correlation between the expression of B3GALT5-AS1 and miR-203 in these 64 colon cancer tissues displayed that the expression of miR-203 was inversely correlated with that of B3GALT5-AS1 in colon cancer tissues (Fig. 5B). miR-203 expression in 15 pairs of primary colon cancer tissues and corresponding liver metastasis tissues used in Fig. 1D was also detected. As displayed in Fig. 5C, miR-203 was significantly upregulated in liver metastasis tissues compared with primary colon cancer tissues. In addition, the expressions of ZEB2 and SNAI2 were measured in the same 15 pairs of primary colon cancer tissues and corresponding liver metastasis tissues. The results displayed that ZEB2 and SNAI2 were both downregulated in liver metastasis tissues compared with primary colon cancer tissues (Fig. 5D, E). The expression of EMT markers E-cadherin and N-cadherin were also measured in these paired primary colon cancer tissues and liver metastasis tissues. As displayed in Fig. 5F, G, E-cadherin was upregulated and while N-cadherin was downregulated in liver metastasis tissues compared with primary colon cancer tissues. These data suggested that miR-203 is increased in colon cancer and further increased in liver metastasis tissues, which is inversely associated with B3GALT5-AS1. ZEB2 and SNAI2 were reduced, and epithelial feature was preferred in liver metastasis tissues.

### B3GALT5-AS1 suppresses colon cancer liver metastasis

**Figure 6:**
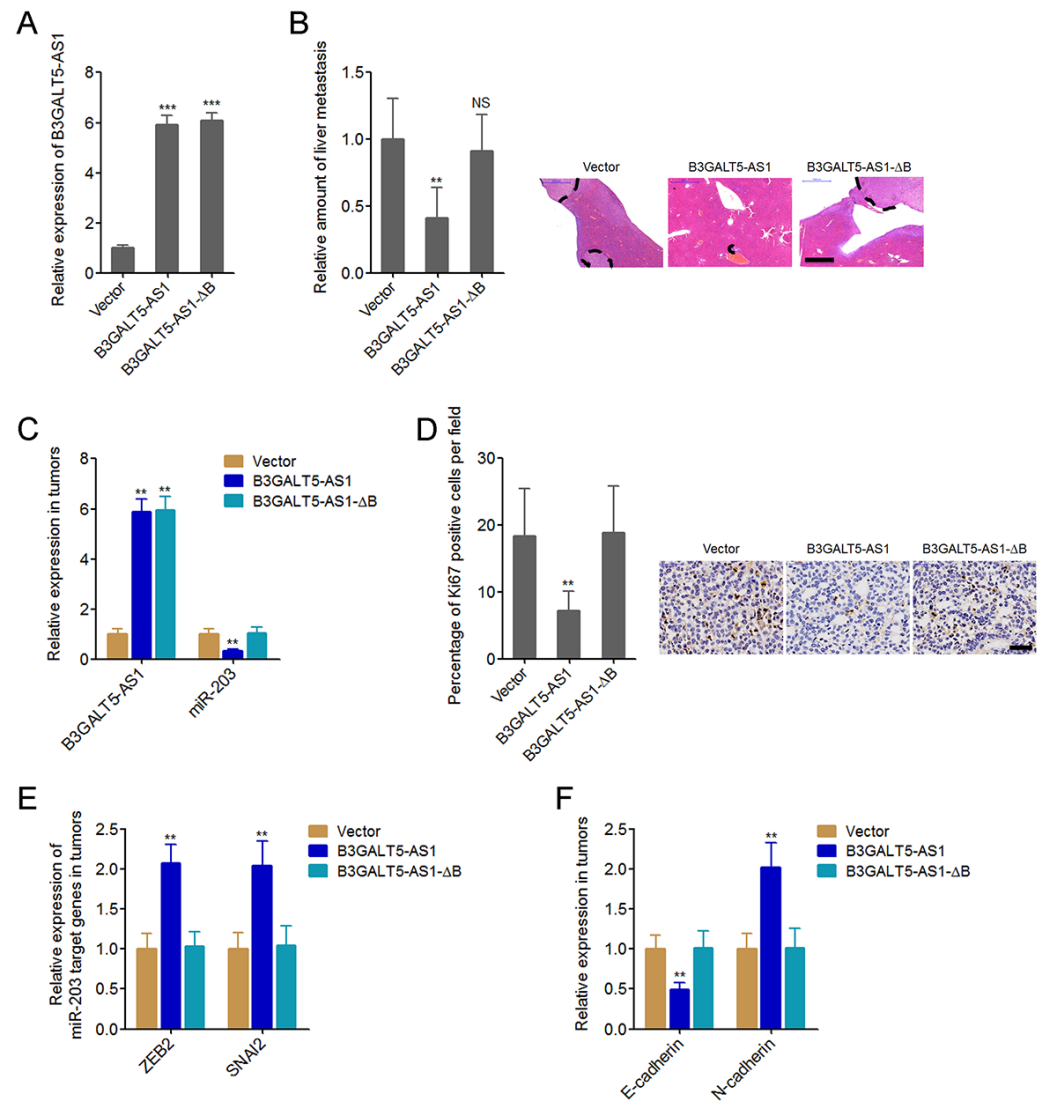
**B3GALT5-AS1 inhibited colon cancer liver metastasis.** (**A**) B3GALT5-AS1 expression in different B3GALT5-AS1 stably overexpressed HCT116 cells clones was measured using qRT-PCR. Data are displayed as mean ± s.d. of three independent experiments. ***P < 0.001, Student’s *t*-test. (**B**) Indicated B3GALT5-AS1 stably overexpressed HCT116 cells were intra-splenic injected to establish liver metastasis. The amount of liver metastatic foci was assessed at the 42^th^ day after intra-splenic injection using HE staining. Scale bars = 1000 μm. (**C**) The expression of B3GALT5-AS1 and miR-203 in liver metastatic foci formed by these indicated B3GALT5-AS1 stably overexpressed HCT116 cells was detected using qRT-PCR. (**D**) Immunohistochemical staining of Ki67 in liver metastatic foci formed by these indicated B3GALT5-AS1 stably overexpressed HCT116 cells. Scale bars = 50 μm. (**E**) The expression of ZEB2 and SNAI2 in liver metastatic foci formed by these indicated B3GALT5-AS1 stably overexpressed HCT116 cells was measured using qRT-PCR. (**F**) The expression of E-cadherin and N-cadherin in liver metastatic foci formed by these indicated B3GALT5-AS1 stably overexpressed HCT116 cells was detected using qRT-PCR. For **B**-**F**, data are displayed as mean ± s.d. of six mice in each group. **P < 0.01, NS, not significant, Mann-Whitney test.

Next, we explored whether B3GALT5-AS1 have effects on colon cancer liver metastasis. The binding sites depleted B3GALT5-AS1 was stably overexpressed in HCT116 cells with a similar overexpression level to B3GALT5-AS1 full length overexpression clone (Fig. 6A). B3GALT5-AS1 stably overexpressed and control HCT116 cells were injected through the spleen to establish liver metastasis model in nude mice. The results displayed that ectopic expression of B3GALT5-AS1 decreased the amount of liver metastatic foci, which was abolished by the depletion of binding sites (Fig. 6B). The expressions of B3GALT5-AS1 and miR-203 were measured in the liver metastatic foci formed by these different HCT116 clones. The results confirmed the overexpression of B3GALT5-AS1 and the downregulation of miR-203 in the liver metastatic foci formed by B3GALT5-AS1 stably overexpressed cells (Fig. 6C). Depletion of the binding sites abolished the effects of B3GALT5-AS1 on miR-203 *in vivo* (Fig. 6C). Proliferation marker Ki67 immunohistochemical staining of the liver metastatic foci displayed that B3GALT5-AS1 overexpression decreased the proportion of Ki67-positive cells, which was abolished by the depletion of binding sites (Fig. 6D). The expressions of ZEB2 and SNAI2 were measured in the liver metastatic foci, and the results displayed that ZEB2 and SNAI2 were upregulated in the liver metastatic foci formed by B3GALT5-AS1 stably overexpressed cells, which were abolished by the depletion of binding sites of B3GALT5-AS1 (Fig. 6E). The expression of EMT markers E-cadherin and N-cadherin were also measured in the liver metastatic foci. The results displayed that E-cadherin was downregulated and while N-cadherin was upregulated in the liver metastatic foci formed by B3GALT5-AS1 stably overexpressed cells, which were also abolished by the depletion of binding sites of B3GALT5-AS1 (Fig. 6F). All these results suggested that B3GALT5-AS1 inhibited miR-203, upregulated ZEB2 and SNAI2, induced EMT, and suppressed colon cancer liver metastasis *in vivo*.

### Depletion of B3GALT5-AS1 promotes colon cancer liver metastasis in a miR-203-dependent manner

**Figure 7:**
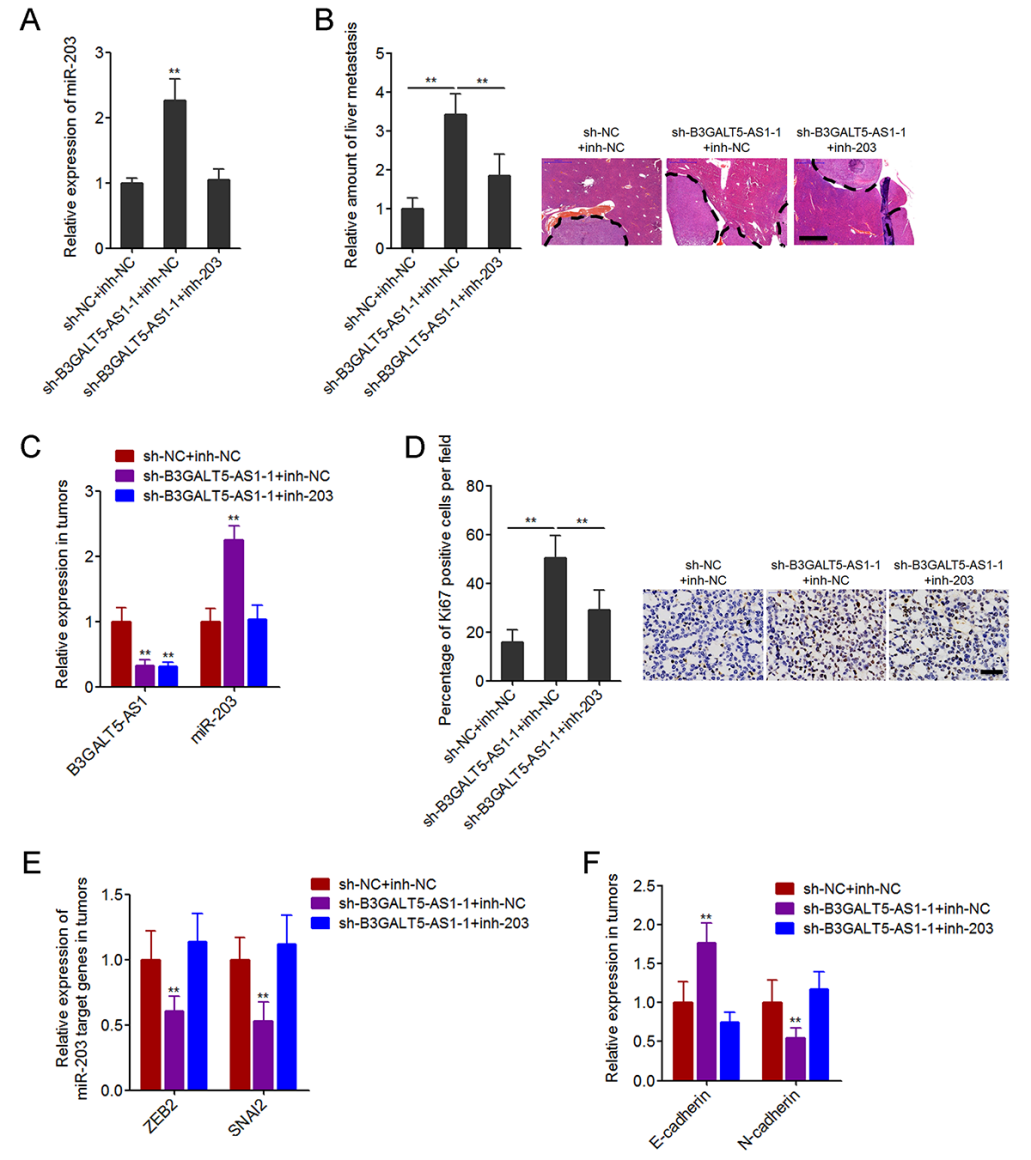
**Depletion of B3GALT5-AS1 promoted colon cancer liver metastasis in a miR-203-dependent manner.** (**A**) miR-203 expression in B3GALT5-AS1 and miR-203 concurrently depleted and control SW620 cells was measured using qRT-PCR. Data are displayed as mean ± s.d. of three independent experiments. **P < 0.01, Student’s *t*-test. (**B**) B3GALT5-AS1 and miR-203 concurrently depleted and control SW620 cells were intra-splenic injected to establish liver metastasis. The amount of liver metastatic foci was detected at the 42^th^ day after intra-splenic injection using HE staining. Scale bars = 1000 μm. (**C**) The expression of B3GALT5-AS1 and miR-203 in liver metastatic foci formed by B3GALT5-AS1 and miR-203 concurrently depleted and control SW620 cells was detected using qRT-PCR. (**D**) Immunohistochemical staining of Ki67 in liver metastatic foci formed by B3GALT5-AS1 and miR-203 concurrently depleted and control SW620 cells. Scale bars = 50 μm. (**E**) The expression of ZEB2 and SNAI2 in liver metastatic foci formed by B3GALT5-AS1 and miR-203 concurrently depleted and control SW620 cells was measured using qRT-PCR. (**F**) The expression of E-cadherin and N-cadherin in liver metastatic foci formed by B3GALT5-AS1 and miR-203 concurrently depleted and control SW620 cells was detected using qRT-PCR. For **B**-**F**, data are displayed as mean ± s.d. of six mice in each group. **P < 0.01, Mann-Whitney test.

To further investigate whether the inhibition of miR-203 mediates the roles of B3GALT5-AS1 in colon cancer liver metastasis, we stably inhibited miR-203 expression in B3GALT5-AS1 stably depleted SW620 cells (Fig. 7A). The constructed cell clones were injected through the spleen to establish liver metastasis model in nude mice. The results displayed that B3GALT5-AS1 knockdown increased the amount of liver metastatic foci, which was attenuated by the inhibition of miR-203 (Fig. 7B). The expressions of B3GALT5-AS1 and miR-203 were measured in the liver metastatic foci formed by these stable clones. The results confirmed the downregulation of B3GALT5-AS1 and the upregulation of miR-203 in liver metastatic foci formed by B3GALT5-AS1 stably depleted cells, and also the inversion of miR-203 in the liver metastatic foci formed by B3GALT5-AS1 and miR-203 concurrently depleted cells (Fig. 7C). Proliferation marker Ki67 immunohistochemical staining of the liver metastatic foci displayed that B3GALT5-AS1 knockdown increased the proportion of Ki67-positive cells, which was attenuated by the inhibition of miR-203 (Fig. 7D). The expressions of ZEB2 and SNAI2 were measured in the liver metastatic foci, and the results displayed that ZEB2 and SNAI2 were downregulated in the liver metastatic foci formed by B3GALT5-AS1 stably depleted cells, which were abolished by the inhibition of miR-203 (Fig. 7E). The expression of EMT markers E-cadherin and N-cadherin were also measured in the liver metastatic foci. The results displayed that E-cadherin was upregulated and while N-cadherin was downregulated in the liver metastatic foci formed by B3GALT5-AS1 stably depleted cells, which were also abolished by the inhibition of miR-203 (Fig. 7F). All these results demonstrated that B3GALT5-AS1 knockdown increased miR-203, downregulated ZEB2 and SNAI2, and inhibited EMT *in vivo*. These data also suggested that B3GALT5-AS1 knockdown promoted colon cancer liver metastasis at least partially via the upregulation of miR-203.

## DISCUSSION

Distant metastasis, particular liver metastasis, is the major cause of colon cancer-related death [4]. However, the critical molecular mechanisms underpinning colon cancer liver metastasis are largely unknown. In the present study, we found a novel regulation axis in the process of colon cancer liver metastasis, which is the induction of EMT by lncRNA B3GALT5-AS1 via repressing miR-203. Our data revealed that B3GALT5-AS1 directly binds to the promoter of *miR-203*, represses *miR-203* expression, upregulates miR-203 targets ZEB2 and SNAI2, induces EMT, and finally suppresses colon cancer liver metastasis. Consistent with the suppressive roles of B3GALT5-AS1/miR-203/ZEB2-SNAI2/EMT in colon cancer liver metastasis, B3GALT5-AS1 is reduced, miR-203 is increased, ZEB2 and SNAI2 are reduced, epithelial marker E-cadherin is increased, mesenchymal marker N-cadherin is reduced in liver metastasis tissues compared with primary colon cancer tissues.

In the liver metastatic foci, the metastasized colon cancer cells undergo MET and regain epithelial phenotype to permit their settlement and proliferation [54]. Our data support this theory. Our *in vivo* liver metastasis assays demonstrated that overexpression of B3GALT5-AS1 induced mesenchymal phenotype of liver metastasized colon cancer cells and inhibited liver metastasis of colon cancer. Depletion of B3GALT5-AS1 induced epithelial phenotype of liver metastasized colon cancer cells and promoted liver metastasis of colon cancer. Therefore, the opposing roles of EMT in early invasion and late settlement of colon cancer liver metastasis processes imply that disease stage-specific therapies are warranted.

Mechanistically, we identified a long interaction region with about 300 nucleotides between the last 300 nucleotides of B3GALT5-AS1 and the promoter of *miR-203*. ChIRP assays revealed the physical binding between B3GALT5-AS1 and the promoter of *miR-203*. Dual luciferase reporter assays and depletion mapping assays revealed that B3GALT5-AS1 inhibited the promoter activity of *miR-203*, which was dependent on the interaction region. Consistently, B3GALT5-AS1 repressed miR-203 expression both *in vitro* and in liver metastasized colon cancer cells *in vivo*, which were also dependent on the interaction region. The inverse correlation between B3GALT5-AS1 and miR-203 expression in colon cancer tissues supported the negative regulation of miR-203 by B3GALT5-AS1.

Furthermore, *in vivo* functional assays revealed that inhibition of miR-203 attenuated the pro-metastatic roles of B3GALT5-AS1 depletion in colon cancer liver metastasis. Except for miR-203, other EMT regulators may also be B3GALT5-AS1 downstream targets, which need further investigation. Excluding EMT, other mechanisms may also mediate the roles of B3GALT5-AS1 in colon cancer cell proliferation, which also need further investigation. Nonetheless, our results suggested that the negative regulation of miR-203 and positive regulation of EMT by B3GALT5-AS1 at least partially mediated the roles of B3GALT5-AS1 in colon cancer liver metastasis.

In summary, we demonstrated that lncRNA B3GALT5-AS1 is reduced in colon cancer tissues, and further reduced in colon cancer liver metastasis tissues. Low expression of B3GALT5-AS1 indicts poor outcome of colon cancer patients. B3GALT5-AS1 inhibits proliferation, promotes migration and invasion, induces EMT, and inhibits liver metastasis of colon cancer cells via repressing miR-203. Our data suggested that B3GALT5-AS1/miR-203/EMT axis may be potential therapeutic target for colon cancer liver metastasis.

## MATERIALS AND METHODS

### Patient and tissue specimens

Sixty-four pairs of primary colon cancer tissues and adjacent normal colonic epithelium tissues, and 15 colon cancer liver metastasis tissues were collected from colon cancer patients with written informed consent who received surgical resection at The First Affiliated Hospital, Sun Yat-sen University (Guangzhou, China). These tissue samples were diagnosed with pathological examination. All resected tissues were immediately snap-frozen in liquid nitrogen and stored at -80 °C until use. The Research Review Board of The First Affiliated Hospital, Sun Yat-sen University reviewed and approved this study.

### Cell culture

The human normal colonic epithelial cell line NCM460 and colon cancer cell lines HCT116, HT-29, LoVo, SW480 and SW620 were acquired from the Institute of Biochemistry and Cell Biology of the Chinese Academy of Sciences (Shanghai, China). NCM460 was maintained in Dulbecco’s Modified Eagle’s Medium (Gibco, Grand Island, NY, USA). HCT116 and HT-29 were cultured in McCoy's 5A Medium (Sigma-Aldrich, Saint Louis, MO, USA). LoVo was cultured in Ham's F-12K Medium (Invitrogen, Carlsbad, CA, USA). SW480 and SW620 were maintained in L-15 Medium (Gibco). These cells were maintained in the above described medium added with 10% fetal bovine serum (Gibco) at 37°C in a humidified incubator with 5% CO_2_.

### RNA isolation and quantitative real-time PCR (qRT-PCR)

Total RNA was isolated from indicated tissues and cells with TRIzol Regent (Invitrogen) according to the manufacturer’s instruction. The isolated RNA was deal with DNase I (Takara, Dalian, China) to get rid of genomic DNA. Next, reverse transcription was carried out using the RNA and the M-MLV Reverse Transcriptase (Invitrogen) following the manufacturer’s instruction. Quantitative real-time PCR (qRT-PCR) assays were performed using SYBR^®^ Premix Ex Taq™ II (Takara) on ABI StepOnePlus Real-Time PCR System (Applied Biosystems, Foster City, CA, USA) following the manufacturers’ protocols. β-actin was employed as an endogenous control for the quantitation of mRNAs and lncRNAs. Primers’ sequences were as follows: for transcript variant 1 of B3GALT5-AS1, 5'-ATTTCACGGATGAGACGAC-3' (forward) and 5'-CCTTGAGAGACGAAGCAC-3' (reverse); for transcript variant 2 of B3GALT5-AS1, 5'-TCACGGATGAGACGACTC-3' (forward) and 5'-AAGGCTTCCAAACACGAAAA-3' (reverse); for E-cadherin, 5'-GCCCCATCAGGCCTCCGTTT-3' (forward) and 5'-ACCTTGCCTTCTTTGTCTTTGTTGGA-3' (reverse); for N-cadherin, 5'-TGGACCATCACTCGGCTTA-3' (forward) and 5'-ACACTGGCAAACCTTCACG-3' (reverse); for ZEB2, 5'-TGAGGATGACGGTATTGC-3' (forward) and 5'-ATCTCGTTGTTGTGCCAG-3' (reverse); for SNAI2, 5'-GGCAAGGCGTTTTCCAG-3' (forward) and 5'-CAGCCAGATTCCTCATGTTT-3' (reverse); and for β-actin, 5'-GGGAAATCGTGCGTGACATTAAG-3' (forward) and 5'-TGTGTTGGCGTACAGGTCTTTG-3' (reverse). For miRNAs quantitation, qRT-PCR was carried out as above described with TaqMan microRNA assays following the manufacturer’s instruction (Applied Biosystems). U6 served as an endogenous control for the quantitation of miRNAs. The quantitation of RNA was calculated with the comparative Ct method.

### Western blot

Total proteins were isolated from tissues or cells using RIPA buffer (Beyotime, Shanghai, China). Identical quantity of protein samples were separated using sodium dodecyl sulfate-polyacrylamide gel electrophoresis (SDS-PAGE). Then, proteins were transferred to nitrocellulose filter membrane (Millipore, Bedford, MA, USA). Next, the membranes were blocked with 5% bovine serum albumin, followed by being incubated with primary antibodies against β-actin (Sigma-Aldrich), E-cadherin (Abcam, Hong Kong, China), or N-cadherin (Abcam). After three washes using TBS buffer, the membranes were incubated with IRdye 700-conjugated goat anti-mouse IgG or IRdye800-conjugated goat anti-rabbit IgG (Li-Cor, Lincoln, NE, USA). Last, immunoreactive bands were detected using an Odyssey infrared scanner (Li-Cor).

### Plasmids construction

For construction of different B3GALT5-AS1 overexpression plasmids, B3GALT5-AS1 full-length nucleotides, 1-1061 nucleotides of B3GALT5-AS1, and 1062-1363 nucleotides of B3GALT5-AS1 were PCR amplified with Thermo Scientific Phusion Flash High-Fidelity PCR Master Mix (Thermo-Fisher Scientific, Waltham, MA, USA). Then, the PCR products were subcloned into the Hind III and Xba I, Hind III and BamH I, or BamH I and Xba I sites of the pcDNA3.1 plasmid (Invitrogen), termed as pcDNA3.1-B3GALT5-AS1, pcDNA3.1-B3GALT5-AS1-∆B, pcDNA3.1-B3GALT5-AS1-B, respectively. The PCR primers’ sequences are as follows: for pcDNA3.1-B3GALT5-AS1, 5'-CCCAAGCTTGACGCGGCGGGCGGCTCC-3' (forward) and 5'-GCTCTAGAAATTTTACTTTTTTTGGAGACAGGG-3' (reverse); for pcDNA3.1-B3GALT5-AS1-∆B, 5'-CCCAAGCTTGACGCGGCGGGCGGCTCC-3' (forward) and 5'-CGGGATCCTATGGAGGTTCTGTTTGCTTCTGCA-3' (reverse); for pcDNA3.1-B3GALT5-AS1-B, 5'-CGGGATCCAAATGTAATGATGTCTTGTGCC-3' (forward) and 5'-GCTCTAGAAATTTTACTTTTTTTGGAGACAGGG-3' (reverse). Empty plasmid pcDNA3.1 was employed as negative control. Two pairs of cDNA oligonucleotides suppressing B3GALT5-AS1 expression were inserted into the SuperSilencing shRNA expression plasmid pGPU6/Neo (GenePharma, Shanghai, China), named sh-B3GALT5-AS1-1 and sh-B3GALT5-AS1-2. The target sites are 5'-GCAAGACAGCGCATTGATTGG-3' and 5'-GCATAAGAGAGACCAACTTGG-3', respectively. A scrambled shRNA was employed as negative control and named sh-NC. The promoter of *miR-203* containing the predicted B3GALT5-AS1 binding sites was PCR amplified using the Thermo Scientific Phusion Flash High-Fidelity PCR Master Mix and subcloned into the Kpn I and Hind III sites of firefly luciferase reporter pGL3-Basic plasmid (Promega, Madison, WI, USA), named pGL3-miR203-pro. The PCR primers’ sequences are as follows: 5'-GGGGTACCTCCTCTCCATCACGACTACT-3' (forward) and 5'-CCCAAGCTTGTTTCTGCTTCTCAGACCCT-3' (reverse).

### Stable cell lines construction

For construction of B3GALT5-AS1 stably overexpressed HCT116 cells, pcDNA3.1-B3GALT5-AS1, pcDNA3.1-B3GALT5-AS1-∆B, or pcDNA3.1 was transfected into HCT116 cells with Lipofectamine 3000 (Invitrogen) following the manufacturer’s protocols. Next, the cells were selected with neomycin for four weeks. For construction of B3GALT5-AS1 stably depleted SW620 cells, sh-B3GALT5-AS1-1, sh-B3GALT5-AS1-2, or sh-NC was transfected into SW620 cells with Lipofectamine 3000 (Invitrogen). Next, the cells were selected with neomycin for four weeks. Recombinant lentiviruses containing miR-203 inhibitor or the control were purchased from GenePharma (Shanghai, China). B3GALT5-AS1 stably depleted SW620 cells were transfected with 2×10^6^ transducing units of miR-203 inhibition lentiviruses and selected with puromycin for four weeks. The stably cell lines were identified by qRT-PCR.

### Cell proliferation assay

Cell proliferation was assessed by Glo cell viability assay and Ethynyl deoxyuridine (EdU) incorporation assay. For Glo cell viability assay, 2,000 colon cancer cells per-well were plated into 96-well plates and maintained for indicated time. At the end of the incubation period, luminescence values were measured with the CellTiter-Glo^®^ Luminescent Cell Viability Assay (Promega) following the manufacturer’s protocol. EdU incorporation assay was carried out using the EdU kit (Roche, Mannheim, Germany) following the manufacturer’s instruction. The results were collected with the Zeiss fluorescence photomicroscope (Carl Zeiss, Oberkochen, Germany) and measured via counting at least ten random fields.

### Cell migration and invasion assays

Cell migration and invasion were evaluated by transwell assays. For transwell migration assay, 40,000 indicated colon cancer cells resuspended in serum-free medium with 1 μg/ml Mitomycin C to repress cell proliferation were seeded into the upper chambers of transwell inserts (Millipore). Medium supplemented with 10% FBS was added to the lower chambers. After incubation for 24 hours, colon cancer cells remaining on the upper membranes were fully removed. The colon cancer cells migrated through the membranes were fixed in methanol, stained using 0.1% crystal violet, and imaged with the Zeiss fluorescence photomicroscope (Carl Zeiss). The results were measured via counting at least ten random fields. Transwell invasion assay was performed with the Cell Invasion Assay Kit from CHEMICON (Millipore) according to the manufacturer’s protocol. The results were analyzed as transwell migration assay.

### Purification of cytoplasmic and nuclear RNA

Cytoplasmic and nuclear RNA was purified with Cytoplasmic & Nuclear RNA Purification Kit (Norgen, Belmont, CA, USA) following the manufacturer’s protocol. The purified RNA was measured using qRT-PCR.

### Chromatin isolation by RNA purification (ChIRP)

ChIRP was carried out using Magna ChIRP RNA Interactome Kit (Millipore) following the manufacturer’s protocol. Anti-sense DNA probes specific for B3GALT5-AS1 were synthesized by Biosearch Technologies. Probes sequences are as follows: 1, 5'-aaactcaaagaaccggcctc-3'; 2, 5'-ggcatctggggtttgagaag-3'; 3, 5'-ttgcatgactttggctcatt-3'; 4, 5'-taagtattgctccagcattc-3'; 5, 5'-gaagatagcctctctgacag-3'; 6, 5'-atacctcttttgacagagct-3'; 7, 5'-ccacctcaaaggatgatcaa-3'; 8, 5'-ttctgcaccttggtctaatc-3'. ChIRP enriched DNA was measured by qRT-PCR to assess *miR-203* promoter enrichment. Primers’ sequences were as follows: 5'-ACTGGGAAGATGGAGGTTG-3' (forward) and 5'-GATGGAAGTGGGCATAGGG-3' (reverse).

### Dual luciferase reporter assay

The constructed firefly luciferase reporter pGL3-miR203-pro was cotransfected with renilla luciferase expression vector pRL-TK into indicated SW620 cells. The different B3GALT5-AS1 expression plasmids were cotransfected with pGL3-miR203-pro and pRL-TK into HCT116 cells. Forty-eight hours after transfection, the luciferase activity was detected by Dual-Luciferase^®^ Reporter Assay System (Promega) following the manufacturer’s protocols.

### Animal study

To establish *in vivo* liver metastasis model, 2×10^6^ indicted colon cancer cells in 100 μL phosphate buffered saline were intra-splenic injected into 6-week old nude mice acquired from Laboratory Animal Center of Sun Yat-sen University (Guangzhou, China). The mice were housed in a temperature and light controlled pathogen-free animal facility with free access to food and water to being allowed to grow for 6 weeks. Then the mice were sacrificed and the livers were resected. The resected livers were fixed in formalin, paraffin embedded, deparaffinized, rehydrated, and antigen retrieved. The amount of liver metastatic foci was counted via hematoxylin-eosin (HE) staining. The sections were incubated with primary antibody specific for Ki67 (Abcam) and horseradish peroxidase-conjugated secondary antibody (Beyotime, Shanghai, China), followed by being visualized with 3, 3-diaminobenzidine. The animal care and use committee of The First Affiliated Hospital, Sun Yat-sen University reviewed and approved the experimental protocols concerning the handling of mice.

### Statistical analysis

Statistical analyses were performed using GraphPad Prism Software (GraphPad Software, La Jolla, CA, USA). Student’s *t*-test, Wilcoxon signed-rank test, Mann-Whitney test, Pearson chi-square test, Pearson’s correlation analysis, or Log-rank test was carried out as indicated. *P*-values < 0.05 were considered as statistically significant.

## SUPPLEMENTARY MATERIAL

Supplementary Figure S1

Supplementary Figure S2

Supplementary Figure S3
