# Supplementary material for: Long noncoding RNA B3GALT5-AS1 suppresses colon cancer liver metastasis via repressing microRNA-203
Source: Aging (Albany NY). 2018 Dec 10;10(12):3662–82. doi: 10.18632/aging.101628 (PMC6326654; doi:10.18632/aging.101628)
Supplement: Supplementary Figure S1 [file aging-10-101628-s001.pdf]

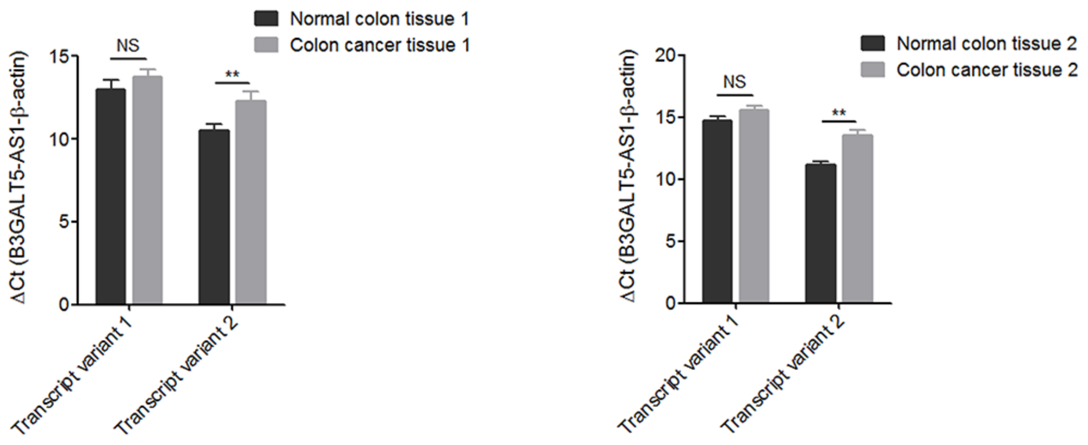

Figure S1. The expression of transcript variants of B3GALT5-AS1 in colon cancer. The expression of B3GALT5-AS1 in 2 pairs of primary colon cancer tissues and adjacent normal colonic epithelium tissues was measured using qRT-PCR. Data are displayed as mean ± s.d. of three independent experiments. **$P < 0.01$, NS, not significant, Student's *t*-test.
