# Supplementary material for: Long noncoding RNA B3GALT5-AS1 suppresses colon cancer liver metastasis via repressing microRNA-203
Source: Aging (Albany NY). 2018 Dec 10;10(12):3662–82. doi: 10.18632/aging.101628 (PMC6326654; doi:10.18632/aging.101628)
Supplement: Supplementary Figure S2 [file aging-10-101628-s002.pdf]

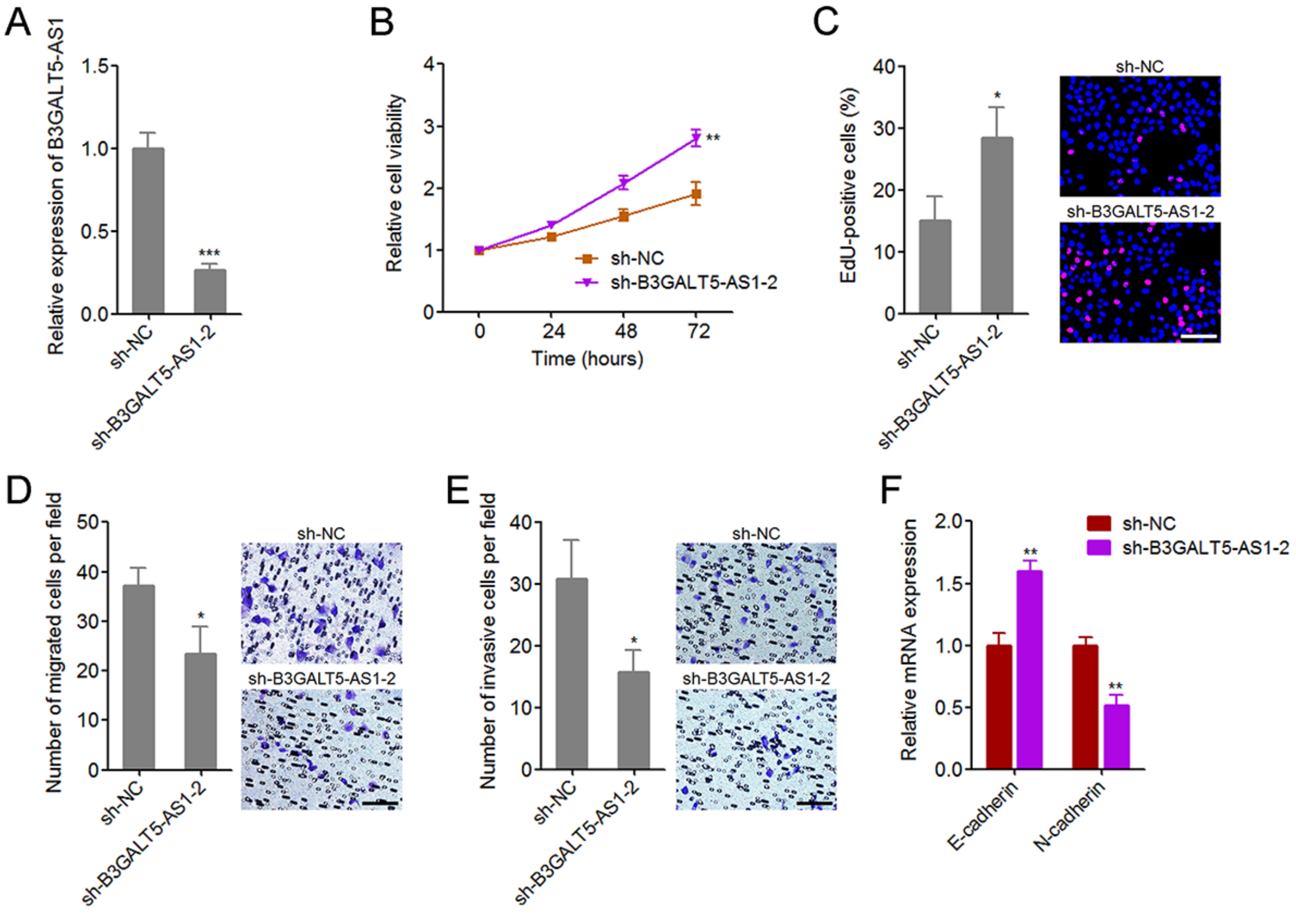

Figure S2. Knockdown of B3GALT5-AS1 promoted proliferation and suppressed migration, invasion, and EMT of NCM460 cells. (**A**) After transient transfection of B3GALT5-AS1 specific or control shRNA into NCM460 cells, the expression of B3GALT5-AS1 was detected using qRT-PCR. (**B**) After transient transfection of B3GALT5-AS1 specific or control shRNA into NCM460 cells, cell viability was detected using Glo cell viability assay. (**C**) After transient transfection of B3GALT5-AS1 specific or control shRNA into NCM460 cells, cell proliferation was detected using EdU incorporation assay. The red color indicts EdU-positive cells. Scale bars = 100 μm. (**D**) After transient transfection of B3GALT5-AS1 specific or control shRNA into NCM460 cells, cell migration was detected using transwell migration assay. Scale bars = 100 μm. (**E**) After transient transfection of B3GALT5-AS1 specific or control shRNA into NCM460 cells, cell invasion was detected using transwell invasion assay. Scale bars = 100 μm. (**F**) After transient transfection of B3GALT5-AS1 specific or control shRNA into NCM460 cells, the expression of E-cadherin and N-cadherin was detected using qRT-PCR. Data are displayed as mean ± s.d. of three independent experiments. *$P < 0.05$, **$P < 0.01$, ***$P < 0.001$, Student's *t*-test.
