# Supplementary material for: Long noncoding RNA B3GALT5-AS1 suppresses colon cancer liver metastasis via repressing microRNA-203
Source: Aging (Albany NY). 2018 Dec 10;10(12):3662–82. doi: 10.18632/aging.101628 (PMC6326654; doi:10.18632/aging.101628)
Supplement: Supplementary Figure S3 [file aging-10-101628-s003.pdf]

A

Promoter of miRNAs	Predicted expect
miR-203	1e-30
miR-34a	5e-20
miR-200b/200a/429	0.32
miR-200c/141	0.097
miR-9	0.003
miR-29a	0.041

B

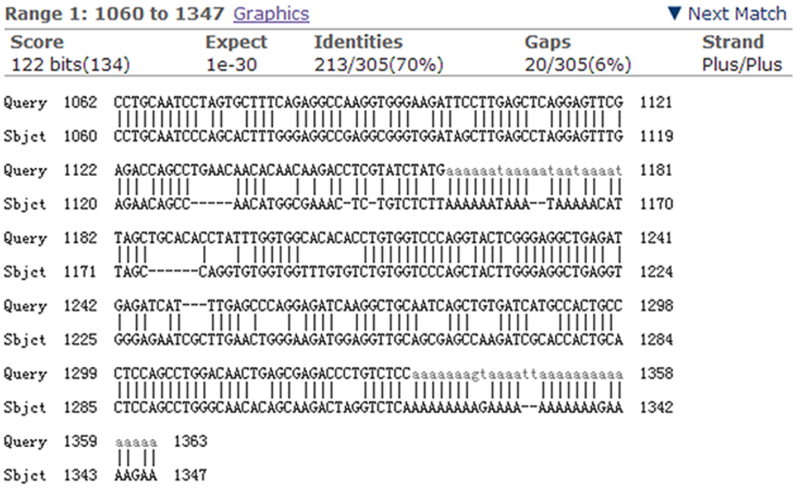

Figure S3. The binding potential between B3GALT5-AS1 and promoters of miRNAs involved in EMT. (**A**) The binding potential between B3GALT5-AS1 and promoters of miRNAs was predicted by Basic Local Alignment Search Tool (BLAST) (https://blast.ncbi.nlm.nih.gov/Blast.cgi). (**B**) Schematic outline of the predicted interaction sites between B3GALT5-AS1 (query) and the promoter of *miR-203* (subject).
